# Synaptic pruning, myelination and the emergence of psychiatric disorders in late adolescence

**DOI:** 10.64898/2026.05.20.726636

**Published:** 2026-05-21

**Authors:** Bruno B. Averbeck, Nicolas Brunel

**Affiliations:** 1 Laboratory of Neuropsychology, National Institute of Mental Health, National Institutes of Health, Bethesda MD, 20892-4415; 2 Department of Computing Sciences, Bocconi University, Milan, Italy

## Abstract

Adolescence is an important developmental period during which there are diverse changes in the brain and behavior. Goal-directed behaviors and the component processes underlying those behaviors improve during adolescence, including working memory, response inhibition, and reinforcement learning. At the same time there is substantial pruning of excitatory connections in prefrontal cortex and ongoing myelination of axons. However, psychiatric disorders also become increasingly prevalent in late adolescence and early adulthood. In this study, we develop computational models that suggest a hypothesis for how the ongoing changes in the brain can give rise to the increased prevalence of psychiatric disorders. We show that both myelination and pruning during adolescence lead to attractor landscapes in which strongly encoded memories, driven by three-factor learning rules that modulate Hebbian plasticity, come to dominate the landscape of brain activity, at the expense of weakly encoded memories. Pruning and myelination lead to large, strong attractors which, if they are related to aversive emotions, can drive intrusive thoughts and compulsions in obsessive compulsive disorder, rumination in depression, and aversive memories in post-traumatic stress disorder. The link between pruning, myelination and the emergence of dominant attractors for emotionally salient memories is well supported by the models. The way these effects map onto forebrain circuits requires more work.

## Introduction

Adolescence is an important developmental period (Larsen and Luna, 2018), during which adult levels of performance are reached on a number of behaviors, including executive function (Wilbrecht and Davidow, 2024). In parallel with these behavioral changes, there are ongoing changes in the brain, that underlie these changes in behavior (Larsen and Luna, 2018). Late adolescence, however, is also a period during which there is an increase in the emergence of psychiatric disorders (Paus et al., 2008; Uhlhaas et al., 2023). There are, at present, few explicit hypotheses that link changes in the brain to changes in behavior, and the increased onset of psychiatric disorders.

Multiple forms of executive function including goal-directed behavior (Welsh et al., 1991), working memory(Fry and Hale, 2000; Gathercole et al., 2004; Montez et al., 2019) response inhibition (Liuzzi et al., 2023) and reinforcement learning (RL) (Palminteri et al., 2016; Nussenbaum and Hartley, 2019; Xia et al., 2021) improve during adolescence (Wilbrecht and Davidow, 2024). Children are better at some forms of exploratory behavior (Gopnik et al., 2015; Sumner et al., 2019; Lloyd et al., 2023). And they have distinct advantages in some forms of learning including language acquisition (Birdsong, 2018) and the large-scale reorganization of circuits that occur in individuals that lose vision at a young age (Cohen et al., 1999). However, directed exploration which requires estimates of uncertainty improves during adolescence(Somerville et al., 2017).

Several neurodevelopmental processes continue through adolescence into adulthood, including synaptic pruning in the cortex (Huttenlocher, 1979; Petanjek et al., 2011), and myelination of long-range axons (Yakovlev and Lecours, 1967; Miller et al., 2012; Grydeland et al., 2013). In early development, neural systems establish connectivity with an abundance of synapses (Rakic et al., 1986; Hensch, 2004). Synapses are subsequently pruned through activity dependent mechanisms to establish adult connectivity and function (Huttenlocher, 1979; Huttenlocher and Dabholkar, 1997; Petanjek et al., 2011; Watanabe and Kano, 2011; Lee et al., 2014; Faust et al., 2021). The number of excitatory connections in prefrontal cortex peaks between the ages of 5 and 8, before decreasing exponentially into adulthood. Estimates suggest that 40–60% of excitatory synapses are pruned. Furthermore, there is evidence that this pruning occurs preferentially on local recurrent synapses, rather than long-range connections (Woo et al., 1997; Kolluri et al., 2005). The number of inhibitory synapses and the number of neurons, on the other hand, remains relatively constant over development (Huttenlocher, 1979; Bourgeois et al., 1994).

There is also ongoing myelination of long-range connections during adolescence including corticocortical, thalamocortical, and corticostriatal axons (Yakovlev and Lecours, 1967). The largest increase in myelination occurs before the age of 5 (Yeung et al., 2014), even in association areas (Grydeland et al., 2013; Baum et al., 2022). However, myelination continues to increase through adolescence and adulthood particularly on axons within the grey matter in association areas. Although it is well-known that myelin increases conduction velocity, it also decreases the variability of synaptic delays (Pelletier and Pare, 2002; Salami et al., 2003; Seidl, 2014; Kato et al., 2020; Lefebvre et al., 2025). Reducing variability in spike timing allows circuits to synchronize dynamic activity across nodes (Brunel, 2000).

Although adolescence is an important developmental period for improvement on a number of behaviors, the emergence of several psychiatric disorders including obsessive-compulsive disorder (OCD), depression, schizophrenia, and substance use disorders (SUDs), also peak in adolescence and early adulthood (Bethlehem et al., 2022; Uhlhaas et al., 2023). The timing of this emergence suggests that changes taking place in the brain during this period increase the risk of developing a psychiatric disorder (Paus et al., 2008). A prominent feature of mood and anxiety disorders including OCD, depression, and post-traumatic stress disorder, is the presence of intrusive thoughts and ruminations. Intrusive thoughts are unwanted, persistent, emotionally distressing thoughts which can become disabling in the context of a psychiatric disorder. Ruminations are internally generated repetitive thoughts with aversive content. These behaviors have often been conceptualized as attractors (Durstewitz et al., 2021). Similarly, in SUDs, drug cues strongly drive craving and compulsive drug seeking (Chow et al., 2025), which may also be a strong attractor. In the context of mental illness, the intrusive thought or rumination is a sequence of activity states in the brain toward which thoughts are strongly drawn, unintentionally, and despite efforts to avoid them. Here, we use the term ‘attractor’ in a loose sense, to refer to a sequence of network states that can be triggered from a very large set of initial conditions. These dynamical attractors can dominate the mental landscape and when they are aversive, it leads to significant distress.

## Methods

We used both rate-based and spiking neural networks to explore the hypothesis that pruning of weak connections can lead to expansion of dynamical attractors, especially those related to strong emotional memories. The implemented networks were developed from previous work, which explored in detail the sequence capacity of these models (Gillett et al., 2020; Gillett and Brunel, 2024).

### Rate-based networks

Rate-based neural networks were governed by the following equations:

$$\tau \frac{dr_{i}}{dt}=-r_{i}+\phi \left(h_{i}\right)+\sigma_{r}\eta ,$$

$$h_{i}=\sum_{j\neq i}^{N}J_{ij}r_{j}.$$

The variable τ is the time-constant of the single-cell dynamics (τ=.010s), $h_{i}$ is the synaptic drive to neuron $i,\;J_{ij}$ is the connection weight from neuron j to neuron i,N is the number of neurons in the network (N=20000), ϕ(h) is the rate transfer function for single neurons, and η is a zero-mean white noise process. This transfer function was given by

$$\phi \left(h\right)=\frac{r_{max}}{2}\left(1+erf\left(\frac{h-\;\theta }{\sqrt{2}\sigma }\right)\right).$$

The variable $r_{\max}$ is the max firing rate of single neurons, θ is the input, h at which the firing rate is half its maximal value, and σ is the inverse gain or slope of the transfer function and erf is an abbreviation for the error function. For all rate network simulations, $\theta =0.22,\;\sigma =0.1,\;r_{\max}=1$.

We used two different scenarios: One with a fixed connectivity matrix, assumed to have been structured by a past learning process; And the other where the connectivity matrix is built iteratively by a learning process. In the first scenario, connection weights in our networks are given by:

$$J_{ij}=J_{S}\frac{c_{ij}}{K}\sum_{s=1}^{S}\sum_{\mu =1}^{P-1}f(\xi_{i}^{S,\mu +1})g(\xi_{j}^{S,\mu }).$$

The variable $c_{ij}$ indexes whether a connection exists $\left(c_{ij}=1\right)$ or not $\left(c_{ij}=0\right)$ from neuron j to neuron i. Networks were sparsely connected. Connections were defined randomly in the network by sampling from a binomial distribution with connection probability given by p. For rate simulations this was p=0.01. K is the total number of connections to each neuron, S is the number of sequences, P is the length of each sequence, and $\xi_{j}^{s,\mu }$ is the activity of neuron j in sequence s for element μ. The functions f and g can be used to implement thresholding. For all simulations in the rate network, these were the identity function. The patterns $\xi_{j}^{s,\mu }$ were randomly sampled from a standard normal distribution and were, therefore, approximately orthogonal to each other within and across sequences.

Sequence recall under noise-free conditions (no perturbation) was tested by setting $h=\xi^{s,1}$. When perturbations were applied, the network was initiated with $r=\phi (\xi^{s,1})+\sigma_{p}\eta$. The value of $\sigma_{p}$ is given in the relevant figures. Correlations between activity in the network and sequences were calculated as:

$$R^{s,\mu }(t)=\frac{\sum_{i=1}^{N}(r_{i}(t)-<r(t){>}_{N})(\xi_{i}^{s,\mu }-<\xi^{s,\mu }{>}_{N})}{\sigma_{r(t)}\sigma_{\xi^{s,\mu }}}$$

For the acquisition and extinction learning simulations, synaptic weights for each sequence are updated at each iteration according to

$$J_{ij}\left(t+1\right)=\left(1-\rho \right)J_{ij}\left(t\right)+\rho J_{s}\frac{c_{ij}}{K}\sum_{\mu =1}^{P-1}f\left(\xi_{i}^{s,\mu +1}\right)g\left(\xi_{j}^{s,\mu }\right),$$

Where $J_{s}$ is the maximum connection strength. This implements the fact that plasticity saturates (Nguyen-Vu et al., 2017). During extinction learning a new sequence is learned that links the cue to no outcome, and the previous sequence that links the cue to the aversive outcome is forgotten. This is done by setting $J_{s}=0$ for the sequence that is being extinguished, because it is not being experienced. Extinction is slower than acquisition (Quirk, 2002), and therefore we used a lower learning rate for extinction. Thus, during extinction and reacquisition, we end up with a superposition of sequences, given by

$$J_{ij}(t)=A_{1}(t)S_{1,ij}+A_{2}(t)S_{2,ij},$$

$$S_{s,ij}=\frac{c_{ij}}{K}\sum_{\mu =1}^{P-1}f\left(\xi_{i}^{s,\mu +1}\right)g\left(\xi_{j}^{s,\mu }\right),$$

where $A_{s}$ indicates the accumulated synaptic strength associated with each sequence.

Three-factor learning rules modulate the strength of synaptic connectivity, driven by Hebbian plasticity, including STDP (Fremaux and Gerstner, 2015; Kusmierz et al., 2017). Memory for events that trigger stress responses engage the glucocorticoid (cortisol) and noradrenergic systems (Shields et al., 2017; Shields et al., 2022), which may modulate synaptic plasticity via three-factor learning rules (Shouval and Kirkwood, 2025). In our networks, when we embedded multiple sequences of different strengths, this was implemented by assuming both the learning rate, ρ and the maximum synaptic strength $J_{s}$ were modulated by a mono-amine neurotransmitter, for example dopamine or norepinephrine (Reynolds et al., 2001). In this case, both were functions of the dopamine concentration, which was assumed to be a function of the emotional intensity of the event. In the present results we only explored the implications of this scaling for the maximum strength of the memory, however, it would be possible to expand this to consider other aspects of this scaling.

Synaptic pruning was implemented by rank ordering the absolute value of all synaptic connections present in the network (i.e. $c_{ij}=1$) and setting a fraction of the lowest rank synapses to 0, $\left(c_{ij}=0\right)$. Specifically, we sorted the synapses such that $J_{ij}^{\vartheta }$ was defined such that $\left\{\left|J_{ij}^{1}\right|<\left|J_{ij}^{2}\right|<\cdots <\left|J_{ij}^{\Pi }\right|\right\}$, where Π is the total number of synapses in the network. We then set all synapses $J_{ij}^{\vartheta }$ to 0, for $\vartheta \leq p_{f}\Pi$, and $p_{f}$ was the fraction of synapses to prune. The remaining synapses were then reweighted, such that the total synaptic strength w in the network was constant before and after pruning. The average input to each neuron is, therefore, kept constant, consistent with previous theory work(Kohler and Widmaier, 1991; Brunel, 2016; Zhang et al., 2019; Iatropoulos et al., 2025):

$$\sum \left|J_{ij}\right|=w.$$

### Spiking networks

We simulated networks of leaky integrate and fire (LIF) spiking neurons. We first simulated a single network of 30,000 neurons to examine sequence recall in a single network. We then simulated two coupled E-I networks with 15,000 neurons each. The number of inhibitory neurons, $N^{I}$, was always $0.25*{\;N}^{E}$. The membrane update equations for the excitatory and inhibitory neurons are given by:

$$\tau_{m}^{E}\frac{dv_{i}}{dt}=-v_{i}+S_{i}^{EE}+S_{i}^{EI}+\sigma_{E}\eta$$

$$\tau_{m}^{I}\frac{dv_{i}}{dt}=-v_{i}+S_{i}^{IE}+\sigma_{I}\eta$$

The variable η was a zero mean gaussian white noise process, and $\sigma_{E}$ and $\sigma_{I}$ were the standard deviations of the excitatory and inhibitory noise, respectively. The post-synaptic potential update equations are given by

$$\tau_{s}^{E}\frac{dS_{i}^{EE}}{dt}=-S_{i}^{EE}+\sum_{j\neq i}^{N^{E}}\sum_{s=1}^{N_{j}^{s}}J_{ij}^{EE}\delta_{j}\left(t-\left(t_{s}+d_{j}\right)\right)$$

$$\tau_{s}^{E}\frac{dS_{i}^{EI}}{dt}=-S_{i}^{EI}+\sum_{j\neq i}^{N^{I}}\sum_{s=1}^{N_{j}^{s}}J_{ij}^{EI}\delta_{j}\left(t-\left(t_{s}+d_{j}\right)\right)$$

$$\tau_{s}^{I}\frac{dS_{i}^{IE}}{dt}=-S_{i}^{IE}+\sum_{j\neq i}^{N^{E}}\sum_{s=1}^{N_{j}^{s}}J_{ij}^{IE}\delta_{j}\left(t-\left(t_{s}+d_{j}\right)\right.$$

Where the variable $d_{j}$ was the synaptic delay associated with pre-synaptic neuron j and $N_{j}^{s}$ is the number of spikes in neuron j. Local delays were always 1 ms. Delays between populations depended on the synaptic delay distribution and are shown in the figures. Each neuron was assumed to have a fixed synaptic delay to all neurons in the population to which it projected, where the delay was given by that neuron’s level of myelination, axonal caliber and distance between nodes of Ranvier (Waxman, 1980). Note that all inhibitory connections were local and therefore all inhibitory synaptic delays were 1 ms. Neuron i fired a spike at time $t_{s}$ if $v_{i}>V_{\mathrm{threshold}}$. After firing a spike, the membrane potential was reset to $V_{\mathrm{reset}}$. Furthermore, neurons had a refractory period and were held at their resting membrane potential, $V_{\mathrm{rest}}$ for 1 ms after firing a spike. Additionally, membrane potentials for excitatory neurons were not allowed to go below the inhibitory reversal potential, which was 10 mv below the reset potential.

Synaptic weights, $J_{ij}$, were constructed using the approach outlined previously (Gillett et al., 2020; Gillett and Brunel, 2024). All structured connectivity was implemented between excitatory neurons. Inhibitory neuron synaptic weights were fixed for all connections. Inhibition and excitation have to be balanced to maintain firing rates within a reasonable range. Excitatory synaptic weights were defined by two steps. First, weights were structured using the same connectivity rule used for rate networks, except incorporating synaptic delays,

$$J_{ij}^{EE*}=J_{S}\frac{c_{ij}}{K}\sum_{s=1}^{S}\sum_{\mu =1}^{P-1}f(\xi_{i}^{s,\mu +d_{j}^{m}})g(\xi_{j}^{s,\mu }).$$

$$d_{j}^{m}=round(\frac{d_{j}}{\tau_{m}^{E}})+1,$$

where the round function rounds its argument to the nearest integer. For spiking networks, the function f and g were given by:

$$f(x)=\left\{\begin{array}{c} q_{f}\;\mathrm{if}\;x>x_{f}\\ 1-q_{f}\;\mathrm{if}\;x\leq x_{f} \end{array}\right.$$

$$g(x)=\left\{\begin{array}{c} q_{g}\;\mathrm{if}\;x>x_{g}\\ 1-q_{g}\;\mathrm{if}\;x\leq x_{g} \end{array}.\right.$$

The variable $c_{ij}$ indicates the presence of a connection ($c_{ij}=1$) or not ($c_{ij}=0$). The probability of a connection between excitatory neurons ($p^{EE}$), inhibitory and excitatory neurons ($p^{EI}$), and excitatory and inhibitory neurons ($p^{IE}$) within a local population, as well as the connection between excitatory neurons between populations ($p^{EEx}$) are given in Table 1. The delay was expressed in units of the membrane time constant, because the sequences evolved on a time-scale set by the membrane time constant. Note that all local connections had 1 ms delays, and therefore the synaptic connectivity in local networks was not affected by myelination. Only long-range connections between areas were affected by myelination. It is difficult to constrain the distribution of spike time delays that would be expected in the human brain. In the mouse, two studies have shown that the distribution of delays depends on myelination (Salami et al., 2003; Kato et al., 2020). These studies suggest that some disruption in myelin can lead to standard deviations of delays on the order of several milliseconds, in thalamocortical circuits. Given the massive scaling of distance from the mouse to the human brain, it seems likely that developmental effects may lead to distributions on the order of 10s of milliseconds. We have explored this range in the results.

Weights were then passed through a threshold linear (relu) nonlinearity, such that only positive weights remained,

$$J_{ij}^{EE}=max(0,\omega J_{ij}^{EE*}-T_{s}).$$

For all simulations, ω=1.0, $T_{s}=-0.0038$. The connections to the inhibitory population, and from the inhibitory population to the excitatory population were fixed for all neurons (i.e. $J_{ij}^{EI}=J^{EI}$ for all i,j), and were set using the mean-field approach outlined in (Gillett et al., 2020). Note that EI connections from inhibitory to excitatory neurons were negative. All other connections weights were positive. Networks were sensitive to inhibition levels such that when inhibition was too high neural activity dropped to low levels and sequence recall failed and when inhibition was too low neural activity diverged and sequence recall failed. Inhibition was not adjusted over pruning as excitatory drive was rescaled and therefore the total excitatory drive was constant, and therefore the amount of inhibition necessary to balance the excitation was also constant, in the network.

For sequence recall in spiking networks, activity was initiated by setting $S^{EE}=\phi^{E}\left(\xi^{s,1}\right)+\eta ,\;S^{EI}=0,\;S^{IE}=<\phi^{E}\left(\xi^{s,1}\right)+\eta {>}_{N^{E}}$. The variable η is Gaussian white noise added to the initial activity to perturb it away from alignment with the first sequence element. When activity was started in only one population, $\xi^{s,1}$ was set to zero for all neurons in population 2, for sequence initiation.

When we pruned synapses in the spiking networks, we pruned only the excitatory synapses. The same procedure was used. We pruned a fraction of the weakest excitatory synapses and then reweighted the remaining synapses such that the total excitatory drive in the network was kept constant. Inhibition was not changed.

For the decoding in the acquisition and extinction experiment, we decoded the representation of the last sequence element. We first calculated the minimum Euclidean distance between the network activity throughout recall and the last element for both the aversive and safety sequences,

$$\Delta_{s}={argmin}_{t}\|r(t)-\xi^{s,P}\|,$$

where s indexed either the aversive or safety sequence. This was then passed through a softmax nonlinearity to normalize values between 0 and 1,

$$p\left(a_{s}|r\right)=exp(-\left(\frac{\Delta_{s}}{\beta }\right))/\sum exp(-\left(\frac{\Delta_{i}}{\beta }\right)).$$

The variable β was set to 10. This scales the slope of the transitions.

## Results

Our goal in this study was to use computational modeling to understand how pruning and myelination, both important developmental processes that lead to improvements in cognition, may also increase the risk of developing psychiatric disorders. We used computational modeling to characterize the conditions under which normal developmental processes that occur in adolescence can lead to the emergence of harmful dynamical attractors with large basins of attraction.

**Fig. 1.**
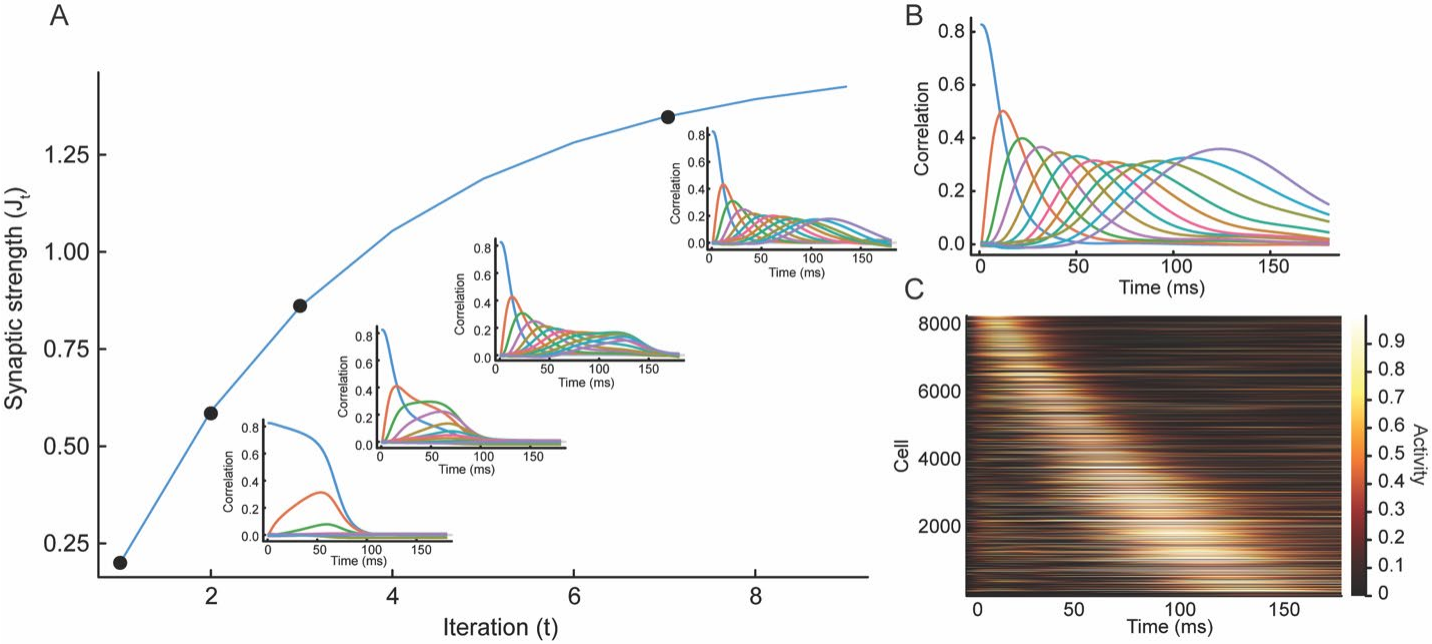
Sequence learning and example activity. For simulation parameters, $J^{s}=1.5,\;\sigma_{r}=0.$ **A**. Sequence learning iterations vs. synaptic strength, and example sequence recall. Network connectivity was trained by exposing the network to the sequence and updating the synaptic connectivity with spike-timing dependent plasticity. Each training iteration strengthened the synaptic connectivity related to the sequence. The ability of the network to generate the sequence autonomously was then probed by starting the network with the first pattern of activity and then letting the network run autonomously. The correlation on the y-axis is the correlation coefficient between the network activity and each pattern in the sequence, where each pattern is a separate curve. The ordinal sequence of peaks represents the networks progression through the sequence. Black dots indicate points at which examples are sampled. The pattern of correlations between network population activity and sequential patterns, with overlaps between adjacent sequence elements, are similar to those measured in prefrontal cortex population activity in the brain (Averbeck et al., 2002). **B**. Sequence recall (synaptic strength = 1.1). **C**. Activity of 8300 example neurons, sorted by time of maximum activity.

We examined the coding of sequences of activity in networks trained with Hebbian plasticity rules (Murray and Escola, 2017; Gillett et al., 2020; Gillett and Brunel, 2024). Exposure of the network to sequential patterns of activity leads to synaptic connectivity changes that encode the sequence. When networks were trained incrementally, synaptic connectivity related to the sequence was gradually strengthened. The networks were then probed for their ability to autonomously generate the sequences by initializing them with the first pattern and then letting them run autonomously. As the synaptic connectivity strengthened, they acquired the ability to autonomously generate the sequences (Fig. 1A). Overlapping sets of single neurons were activated as the sequences unfolded (Fig. 1B, 1C).

**Fig. 2.**
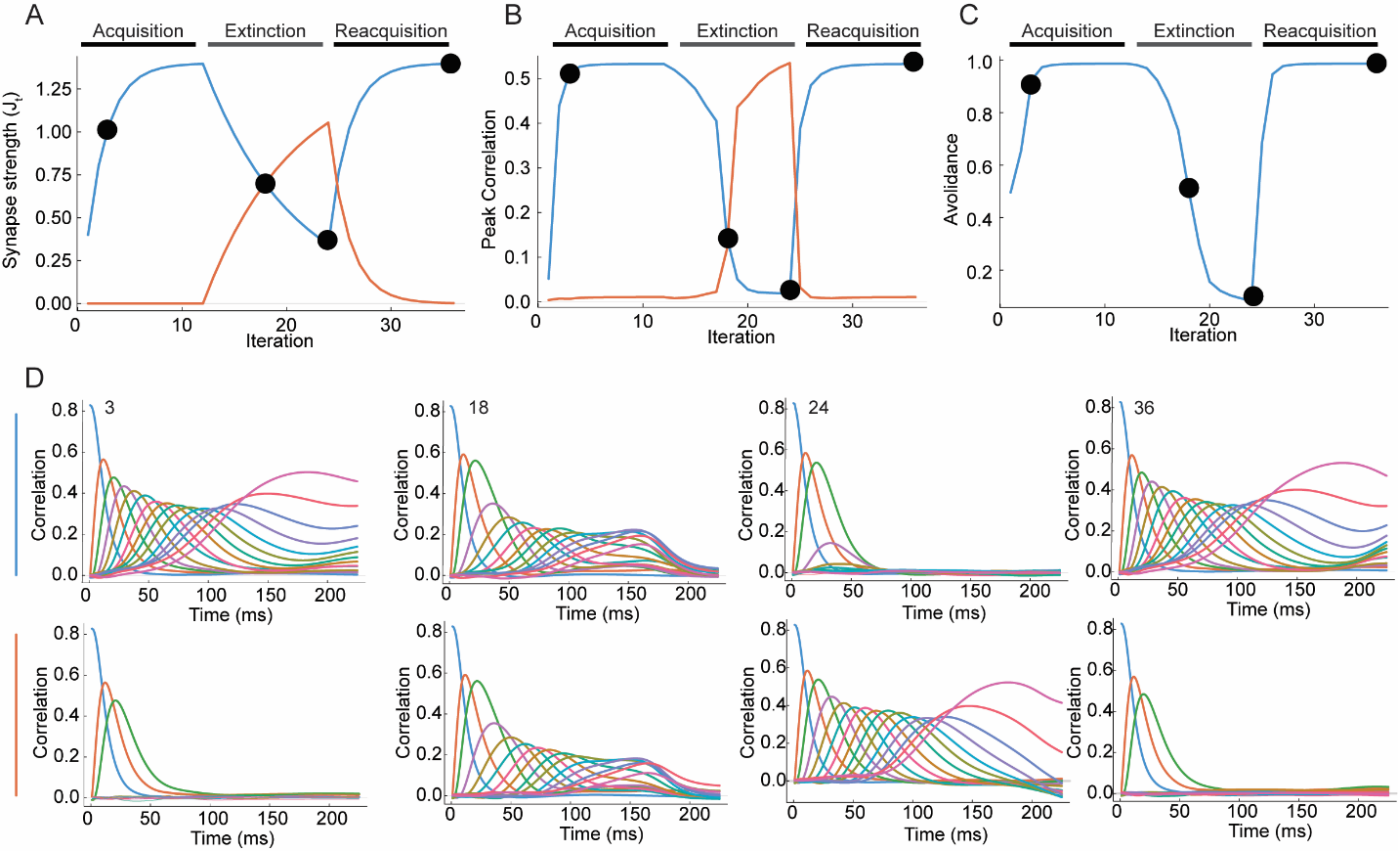
Acquisition, extinction, and reacquisition of sequential activity. For simulation parameters, $J^{s}=1.4,\;\sigma_{r}=0$. During acquisition and reacquisition of a fear memory, ρ=0.4. During extinction (i.e. blue curve during extinction and red curve during reacquisition), ρ=0.11. **A**. Acquisition of sequence representing Pavlovian association of cue and aversive outcome. Blue curves are association of cue (first three sequence elements) with aversive outcome (final element). Red curves are association of the same cue with no outcome. 1–12 are initial acquisition, 13–24 are extinction, and 25–36 are reacquisition. Black dots indicate points at which sequence recall examples are shown in Panel D. **B**. Peak correlation with the last sequence element during sequence recall. Blue curve indicates correlation with aversive association, red curve indicates correlation with safety association. **C**. Avoidance behavior, decoded from population neural activity in the last sequence element representing either predicted aversive or safety outcomes. **D**. Example sequence recall taken from points indicated by black dots in panels A-C. Top row, are for aversive associations, and bottom row are for safety associations. Note that the first three patterns correspond to the cue and are the same in both rows. The inset numbers correspond to the black dots indicated in panels A-C.

Networks in the brain generate sequential patterns of activity in response to sensory stimuli and predicted outcomes (Cunningham and Yu, 2014). Here we explored this as a model for learning and extinction of aversive associations. For disorders including anxiety and OCD, extinction training is an effective treatment. During extinction training in anxiety disorders, a cue that ostensibly predicts a negative outcome is followed by no outcome. In OCD presentation of a cue that normally would drive execution of a ritual is followed by inhibition of the ritual. Therefore, we next explored the ability of the networks to model Pavlovian fear conditioning, extinction, and reacquisition, as an example of these processes. Networks were first trained to associate a cue with an aversive outcome (Fig. 2A). (Note that our states are arbitrary patterns of population activity. However, patterns of activity in the amygdala, for example, represent appetitive and aversive states(Tang et al., 2024).) The cue was modeled as the first three patterns of activity, and the aversive outcome as the remaining patterns in the sequence, similar to the situation in which an aversive outcome is presented at cue termination. Exposing the network to patterns related to the cue and outcome led the networks to acquire the Pavlovian association (Fig. 2D), where the association is reflected in the network autonomously generating a sequence that links cue states (the first three sequence elements) to negative association states (the later elements of the sequence). When we examined the correlation between activity in the network, and the last pattern in the sequence, reflecting the learned association, we found that this correlation quickly increased during initial acquisition (Fig. 2B). Further, when we used network activity to predict avoidance behavior, by decoding the last element of the sequence, we found that it quickly increased during acquisition, reaching a peak after only a few exposures (iterations; Fig. 2C).

Next, the network underwent extinction training. It is known from behavioral work that extinction learning does not erase the aversive memory (Bouton et al., 2021). Rather a new memory is learned, representing the lack of an aversive outcome, or a safety outcome (Macdonald et al., 2025). During extinction training, the network was exposed to the same cue, but the cue was not followed by an aversive US. Rather the cue was followed by no outcome. At the same time, synaptic connectivity related to the aversive outcome association slowly decayed (Fig. 2A). When the network was run autonomously, and the last pattern of activity generated by the network was compared with the aversive and safety outcome patterns, we found that the network shifted during extinction training to generate the pattern related to the safety outcome, and not the aversive outcome (Fig. 2B, D). Similarly, when we predicted avoidance behavior, it was extinguished during extinction training (Fig. 2C).

During extinction the safety prediction came to dominate, and the network generated patterns of activity that predicted a neutral outcome. However, it could be seen that the underlying synaptic connectivity related to the aversive prediction had only partially decayed (Fig. 2A). Thus, when the network was re-exposed to aversive cue-outcome associations, it quickly began to generate patterns related to the aversive outcome (Fig. 2B, D). We then allowed the network to reacquire the aversive association, by running more trials with an aversive outcome, and found that the network quickly reacquired the aversive association.

### Three-factor learning, synaptic pruning and attractor dynamics

**Fig. 3.**
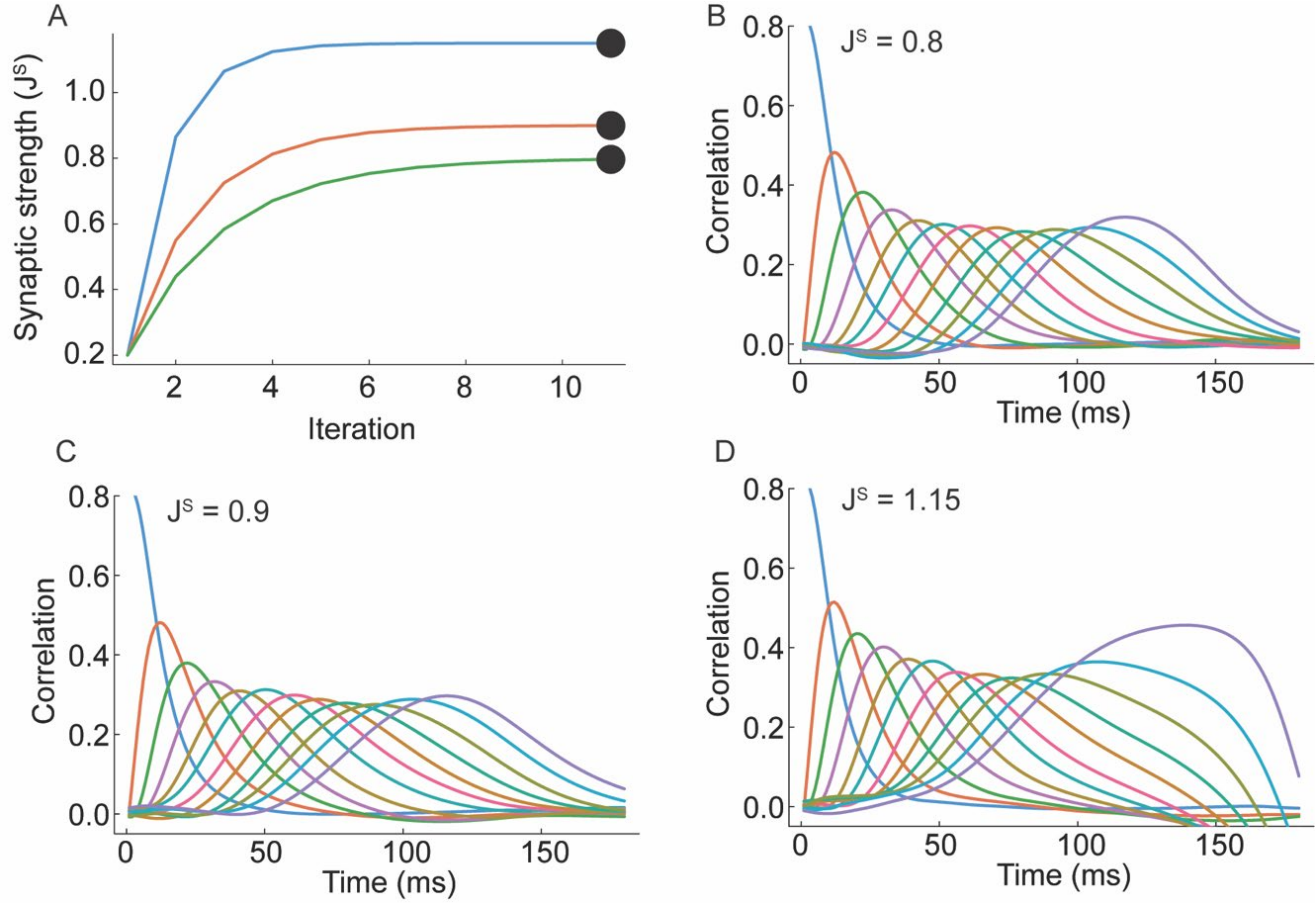
Three factor learning and synaptic strength. **A**. Example simultaneous learning of three different sequences with different dopamine neuromodulation. Neuromodulation affects the rate of learning and the final strength of the sequence. **B**. Sequence recall of a sequence with final overall synaptic strength of $J^{S}=0.8,\;\rho =0.4$. **C**. Sequence recall of a sequence with final overall synaptic strength of $J^{S}=0.9,\;\rho =$ 0.5. **D**. Sequence recall with final overall synaptic strength of $J^{S}=1.15,\;\rho =0.7,\;\sigma_{r}=0$.

Next, we examined the effects of three-factor learning on sequence representation. In several systems, it has been shown that neuromodulators, for example norepinephrine, affect Hebbian learning by gating or modulating plasticity (Fremaux and Gerstner, 2015). When neuromodulators are at low concentrations there is no Hebbian learning, and when concentrations are increased, Hebbian learning is stronger. We used three-factor rules to simulate sequence learning under different levels of emotional intensity, where the emotional intensity of the event drove different levels of neuromodulator release. More specifically, we trained networks on three sequences and implemented three-factor learning by modulating both the rate and asymptotic strength of learning of each sequence differentially (Fig. 3A). When we examined sequence recall in these networks, all three sequences could be recalled in this example under noise-free conditions, when the networks were started with the first pattern of each sequence (Fig. 3B-D). This shows that networks can be trained with multiple sequences, where each sequence has a different strength of representation driven by the interaction of the concentration of the neuromodulator and the Hebbian learning rule.

**Fig. 4.**
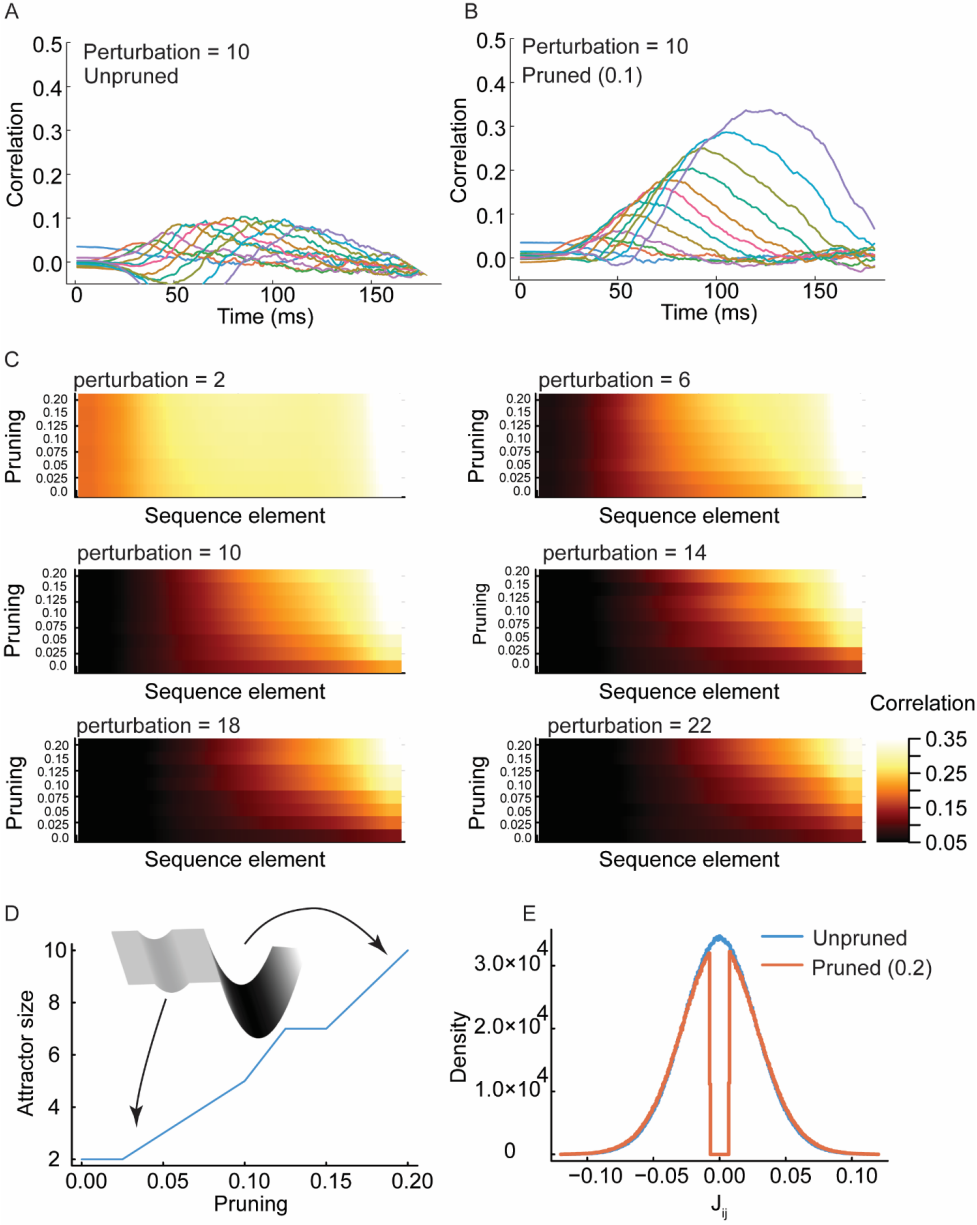
Sequence recall in pruned and unpruned networks with final overall synaptic strength of $J^{S}=1.15,\;\sigma_{r}=0.3$. **A**. Sequence recall in unpruned network when initial pattern of activity is perturbed by adding Gaussian white noise with a standard deviation of 6 to the first pattern. **B**. Recall of the same sequence in a network in which 10% of the weakest connections have been pruned, and the remaining connections have been homeostatically rescaled to maintain the same total strength of synaptic inputs. **C**. Averages across a series of perturbation strengths ($\sigma_{p}$) and levels of pruning, indicated by the fraction of pruned synapses for each row. Correlation indicates the peak correlation of each element of the sequence. Plots are averages over N = 96 random networks and perturbations. **D**. Pruning vs. attractor size. Attractor size was estimated as the maximum perturbation at which the final sequence element exceeded a correlation of 0.2. Note in general that increased pruning consistently increased the strength of the sequence recall across levels of perturbation. Insets are cartoons to indicate that increased pruning increases the size of the dynamical attractor. **E**. Example distribution of individual connection strengths, $J_{ij}$. Not that the pruned curve has more strong connections, but the effect across the distribution is subtle.

Next, we examined the effect of synaptic pruning on recall and the size of attractor basins in networks trained with three sequences with different levels of final synaptic strength. Thus, networks first learned three sequences with different levels of neuromodulation (Fig. 3), with their full synaptic connectivity. We then pruned the weakest connections in these networks and used homeostatic synaptic scaling to strengthen the remaining synapses (Chung et al., 2017; Li et al., 2017) such that the total excitatory and inhibitory synaptic drive in the network was constant (Fig. 4E).

We first examined sequence recall of the strongest sequence, with a final synaptic strength of J^S^ = 1.15. To examine the size of the attractor basin, we perturbed the initial pattern of activity with Gaussian white noise, with different standard deviations (Fig. 4C). Thus, rather than start the network perfectly aligned with the first pattern of activity in the sequence, we started the network with a pattern that deviated from the first pattern, with the size of the deviation given by the standard deviation of the white noise. If the network still recalled the sequence, even though it was not started aligned with the first element, the initial pattern was in the basin of attraction for that sequence. By examining whether the sequence could be recalled with different levels of noise, we could characterize the size of the basin of attraction for each sequence, for different levels of pruning. Networks that recalled sequences when started with a pattern that was far from the first pattern had large basins of attraction, and networks that failed to recall sequences when started far from the first pattern had smaller basins of attraction.

Sequence recall was weaker in unpruned networks (Fig. 4A) and more robust in pruned networks (Fig. 4B) when the initial activity was perturbed with noise. We summarized recall performance by assessing the peak correlation of each sequence element and plotting this as a function of pruning, averaged across 96 networks (Fig. 4C). Across a range of perturbations and levels of pruning, more pruning led to stronger recall at each noise level (Fig. 4C). Thus, pruned networks had larger basins of attraction. When we characterized whether recall of the last element exceeded 0.35 (as an arbitrary threshold to summarize the results) across noise levels, we found that the size of the basin of attraction (estimated as the noise level at which recall exceeded 0.35) increased as the networks were further pruned (Fig. 4D). Thus, pruning increased the basin of attraction for the strongest sequence.

**Fig. 5.**
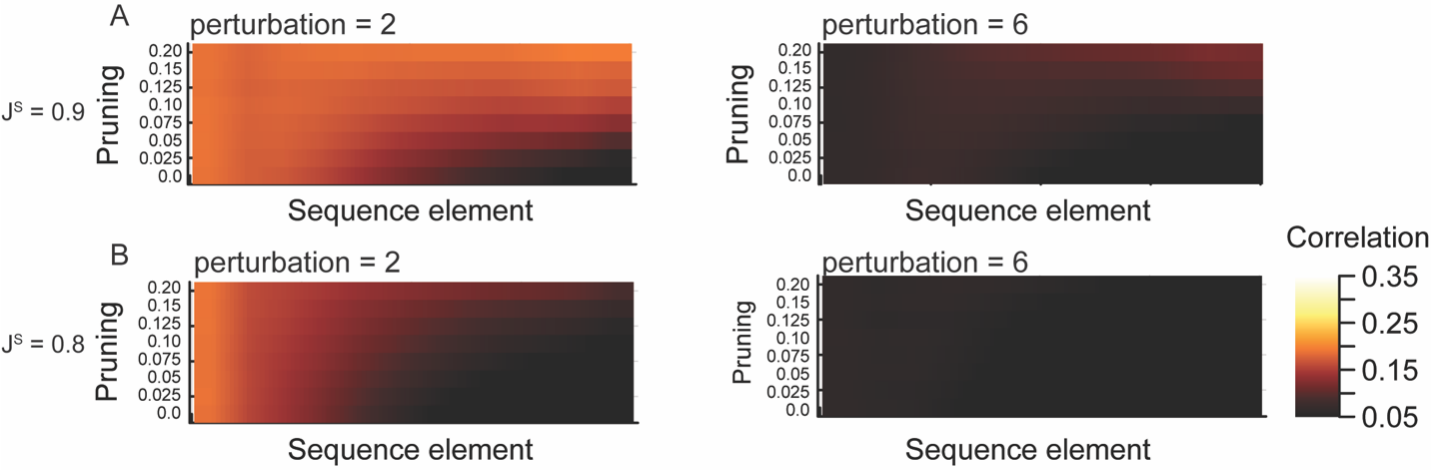
Average recall of sequences with weaker synaptic strength. Same parameters as Figure 4. **A**. Left panel shows recall with low perturbation and different levels of pruning for sequence with overall synaptic strength of $J^{S}=0.9$. Right panel shows the same with higher perturbation. **B**. Same as A for sequence with overall strength of $J^{S}=0.8$.

When we examined sequence recall for the weaker sequences (Fig. 5), we found that, while pruning did increase the strength of recall for the weakest perturbations (Fig. 5A, B, left), sequence recall was not robust at stronger perturbations. Thus, there was minimal recall for even moderate levels of noise (Fig. 5A, B, right). The basins of attraction, therefore, for the weaker sequences were limited. Overall, pruning led to a substantial increase in the basin of attraction for the stronger sequences, such that activity could be started far from the first element, and the sequence was still recalled. Pruning did not lead to robust sequence recall, or expansion of the basin of attraction, for weaker sequences.

### Spiking networks, sequence recall and the effect of myelination

Next, we moved from rate networks to excitatory and inhibitory (E/I) spiking networks. E/I networks allow us to restrict pruning to excitatory connections, consistent with biological pruning in prefrontal cortex (Petanjek et al., 2011). They also allow us to model the effect of myelination on long-range cortical-cortical connections, because spike latency effects can be explicitly modeled.

**Fig. 6.**
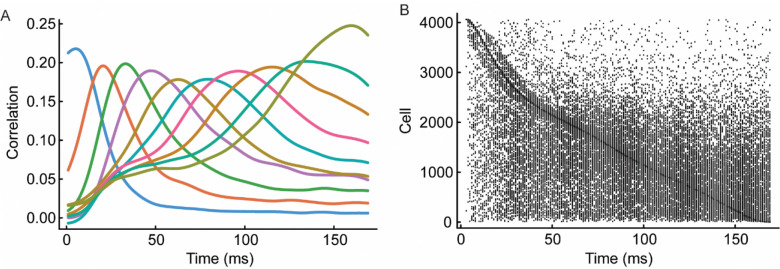
Sequence recall in a network of excitatory and inhibitory spiking neurons. **A**. Sequence recall in network. **B**. Activity of excitatory neurons in the network sorted by time of peak activity in each neuron.

**Fig. 7.**
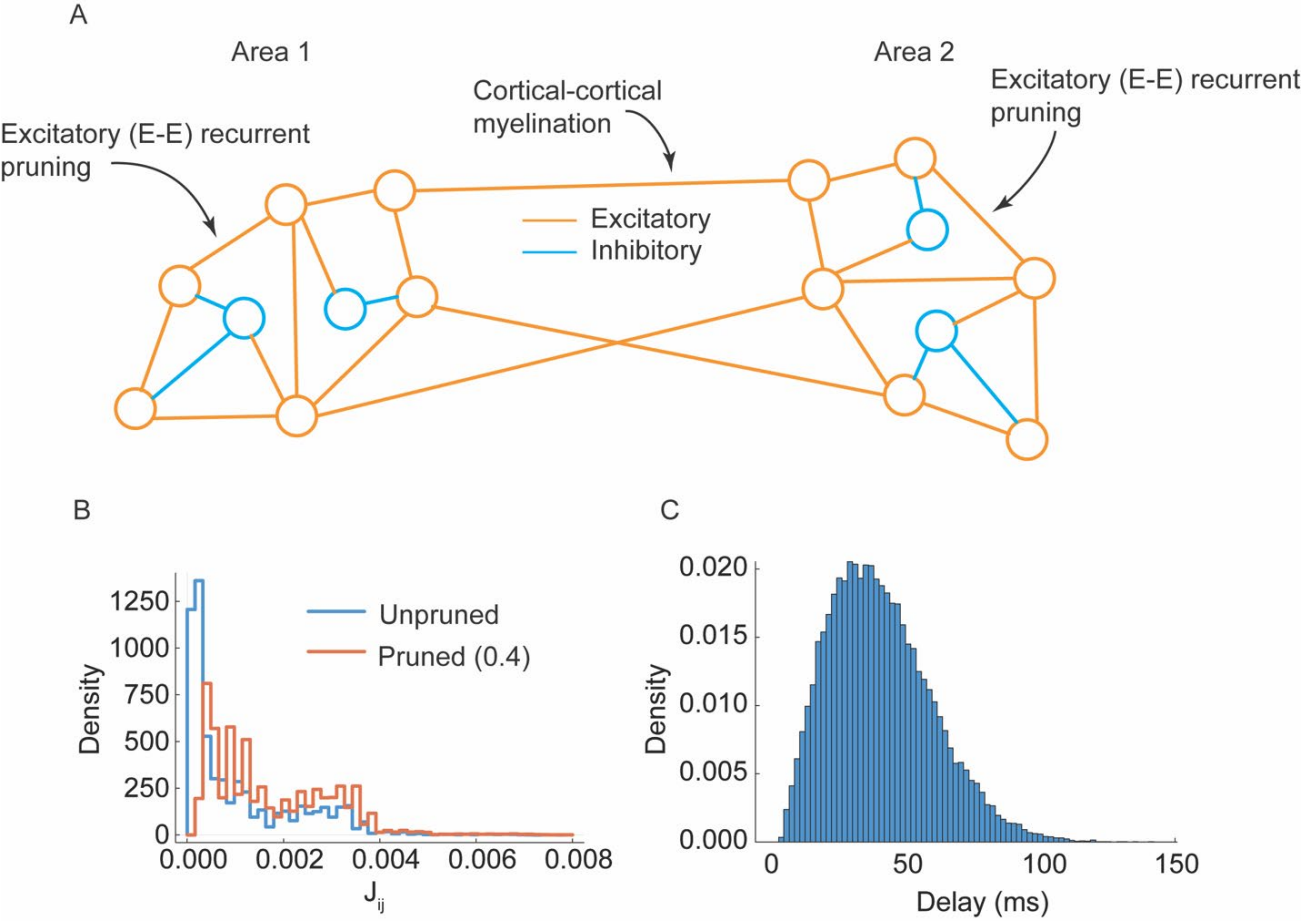
**A.** Network to test effects of pruning and myelination in connected populations of spiking neurons. Each area has N = 15000 excitatory neurons and N = 3750 inhibitory neurons. All inhibitory connections are local. Cortical-cortical connections are excitatory. Local circuit connections are assumed to be fast (i.e. delay = 1 ms). Myelination only affects the distribution of delays on cortical-cortical connections. Pruning was restricted to local E-E connections. E-E connections between populations were not pruned. E-I and I-E connections were not pruned. Weight distributions undergo homeostatic synaptic rescaling to maintain strength of excitatory inputs to each neuron. Note that cortical-cortical connections are bidirectional. Population 1 sends excitatory connections to population 2 and population 2 sends excitatory connections to population 1. Myelination affects delay distributions in both directions. **B.** Example distribution of local recurrent excitatory connections before and after pruning. **C.** Example distribution of delays for long-range excitatory connections, between populations, with location parameter = 30 ms.

We first examined sequence recall in a single network of E/I spiking neurons and replicated previous results that showed robust recall in these networks (Fig. 6). Next, we built a parietal-frontal network with two populations of neurons (Fig. 7). All inhibitory connections were restricted to the local population. Excitatory connections were both local (recurrent) and long-range. Long-range connections were bidirectional such that parietal cortex sent connections to prefrontal cortex, and prefrontal cortex sent return connections to parietal cortex. Local connectivity in all networks had 1 ms delays. Pruning in these networks (e.g. Fig. 7B) was restricted to excitatory connections within each population. Neither long-range connections nor inhibitory connections were pruned. The effect of myelination was modeled as changes in spike time delay distributions on long-range connections (e.g. Fig. 7C). Increasing myelination increases conduction velocity between areas. More relevant to our simulations, however, myelination decreases variability in synaptic delay distributions. Thus, we simulated long-range connections with several distributions of synaptic delays.

**Fig. 8.**
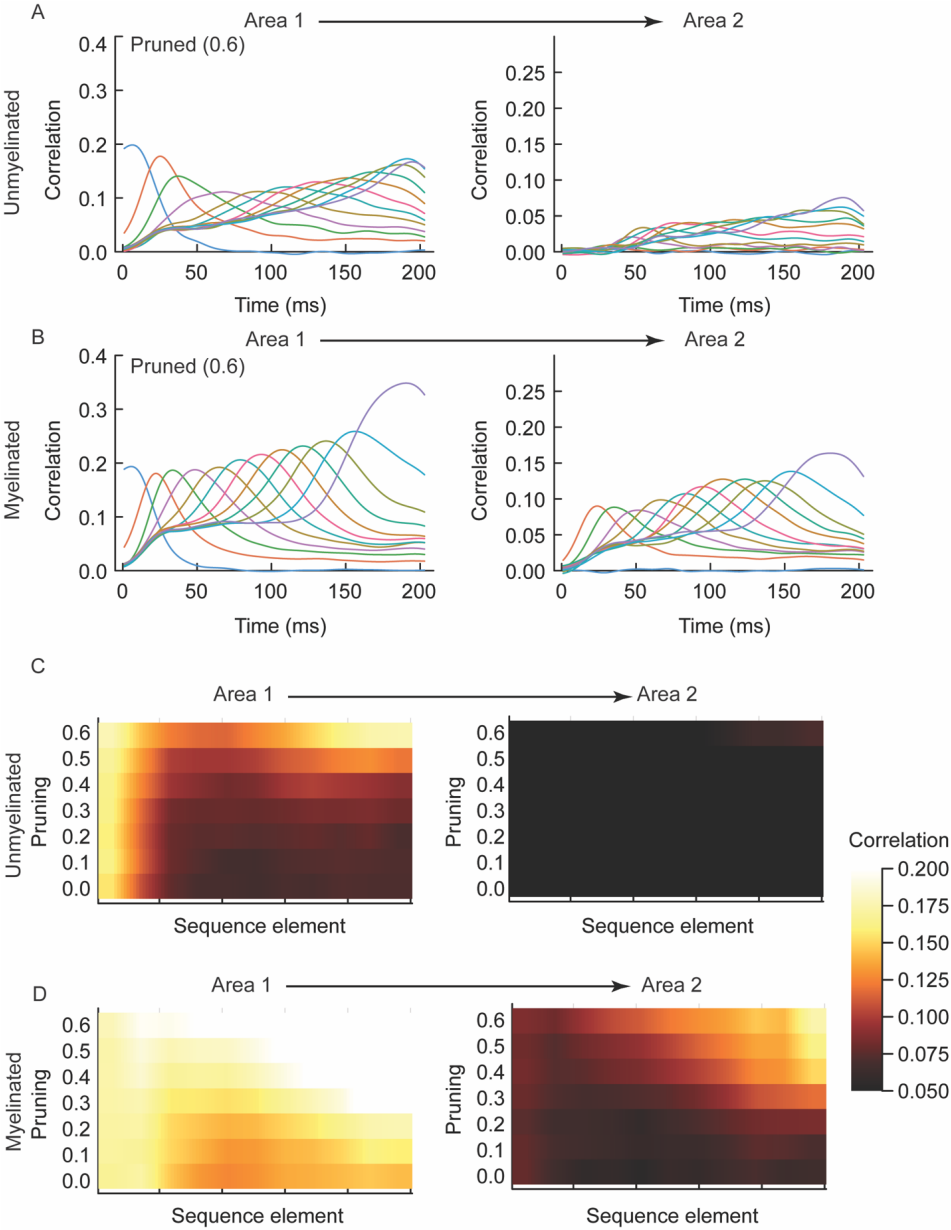
Sequence coupling. Plots are averages over 12 iterations. **A**. Average sequence recall in population 1 and 2 when the sequence activity is started in only population 1 with 30 ms modal average synaptic delay distribution. Sequence activity in population 2 has to propagate from population 1. Pruning = 0.6. Network activity was perturbed with $\sigma_{p}=6$ in both populations. Note that arrow indicates propagation of sequence from population 1 to 2, but population 2 sends feedback connections to population 1. **B.** Same as C for 1 ms delays between all neurons, including long-range connections. Pruning = 0.6. **C.** Peak correlation of sequence elements in population 1 (left panel) and 2 (right panel) with 30 ms modal delay distribution for different levels of pruning. **D.** Same as panel C for 1 ms delays.

We examined propagation of sequences across populations, by starting a sequence in only one population (e.g. parietal cortex), and examining whether the second population (e.g. prefrontal cortex) entrained to the first population (Fig. 8). When we compared sequence coupling between networks with broad synaptic delay distributions on long-range connections (mode = 30 ms, Fig. 7C) to networks with highly constrained, 1 ms delays on long-range connections, we found that coupling was more robust in myelinated networks with narrow delay distributions (Fig. 8A, 8B, right panel). Interestingly, because of the feedback connections from prefrontal to parietal cortex, representations in the networks in which the sequences originated (parietal cortex) also showed stronger sequence representations, even though connectivity and pruning was identical within local networks (Fig. 8A, 8B, left panel). The only difference between the networks was the distribution of delays on long-range connections. Local connections in both networks had the same synaptic connectivity, delays, and pruning. When we compared coupling across areas in these networks for a range of pruning levels, we found that coupling was consistently robust in the network with 1 ms delays, compared to the network with a broad delay distribution (Fig. 8C, 8D). Pruning, however, further strengthened coupling. Thus, both pruning and myelination increase sequence coupling across networks of neurons.

**Fig. 9.**
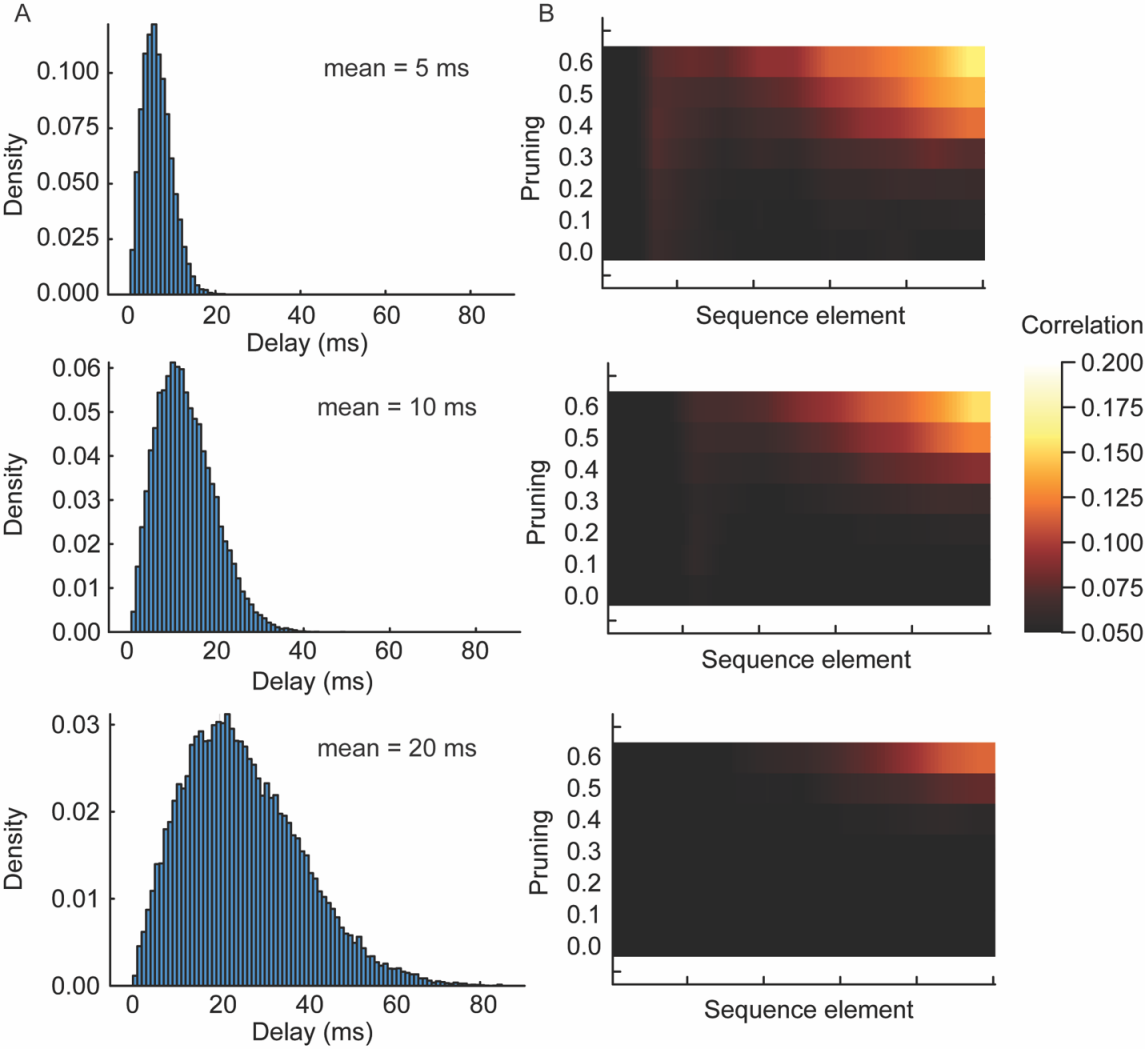
Average sequence recall in population 2 for different delay distributions. All other parameters are the same as Figure 9. **A**. Delay distributions. **B**. Average sequence recall in population 2 at different pruning levels, for each delay distribution.

When we examined various cortical-cortical synaptic delay distributions (Fig. 9A), we found that sequence coupling to prefrontal cortex was consistently weaker as delay distributions became more variable (Fig. 9B). Thus, myelination leads to increased coupling of sequential activity across areas connected by long-range connections in which myelination decreases variability in delay distributions.

## Discussion

Here we have shown that synaptic pruning in rate networks, and excitatory synaptic pruning and increased myelination in spiking networks lead to networks in which emotionally intense memories, with strong synaptic connectivity, can dominate attractor landscapes. While the fractional change in excitatory connections during adolescence (Petanjek et al., 2011) is larger than the fractional change in myelination (Baum et al., 2022) during the same period, both contribute to changing the attractor landscape. Emotionally intense memories create strong synaptic connectivity through three-factor learning rules (Reynolds and Wickens, 2002; Pawlak et al., 2010; Fremaux and Gerstner, 2015). Before synaptic pruning, memories for sequences encoded with both strong and weak synapses can be recalled when they are triggered by a cue which is well-aligned with their initial elements. Following pruning, however, activity patterns far from the initial patterns of sequences that were initially encoded with strong synaptic connectivity, drive recall of those sequences. Less strongly encoded sequences, however, are not well-recalled when activity patterns are not well-aligned. Thus, the attractor basin for strongly encoded sequences is expanded, at the expense of less-strongly encoded sequences, through synaptic pruning.

Myelination affects long-range connections including cortical-cortical (modeled here), thalamocortical, and corticostriatal axons. Myelination increases conduction velocity, which decreases synaptic delays between areas (Salami et al., 2003; Kato et al., 2020). More importantly for our current results, however, myelination also decreases the variability of delays between areas (Pelletier and Pare, 2002; Seidl, 2014). We found that cortical-cortical connections with narrow and short synaptic delay distributions led to stronger coupling of sequences in target areas, and stronger recall within areas in which sequence recall originated. Thus, increased myelination leads to connected networks across which sequential memories couple more effectively. This effect, in combination with pruning, leads to networks in which strongly encoded memories can dominate the attractor landscape and couple throughout connected networks including cortical-striatal-thalamocortical loops. While both pruning and myelination can contribute to the increase in executive function seen during adolescence, they can also unmask embedded emotionally intense memories, which can come to dominate attractor landscapes.

Previous modeling work has shown that synaptic pruning can lead to networks that are more robust to noise, when they are trained on working memory and reinforcement learning tasks (Averbeck, 2022). This study showed that pruned networks trained on working memory tasks were resistant to distraction, modeled as a perturbation of neural activity, during delay periods. When these networks were perturbed away from mean trajectories that encoded working memory for cues during delay periods, they were pulled back towards the mean trajectories and accurately maintained items in memory. Thus, perturbations decayed over time. In addition, pruning these networks during training led to connectivity distributions like those obtained in the networks in the present study with few small connections (as these were pruned away) and more strong connections. These modeling results were followed-up by analyses of longitudinal EEG data, which attempted to characterize whether variability, which is an intrinsic perturbation, in single-trial evoked EEG data in adolescents also showed evidence for stronger attractor dynamics (Liuzzi et al., 2023; Andujar et al., 2026). These studies found that variability in evoked activity decayed back to mean trajectories faster when participants carried out this task in their late (18 y.o.) vs. early (12 y.o) teens. While pruning may primarily drive the effects seen in the EEG data, myelination may also contribute, given the results of the present study.

Previous theoretical studies have addressed how synaptic pruning affects the properties of networks that store information in their connectivity matrix. Random pruning of synaptic connectivity (independent of synaptic strength) increases the efficiency of memory storage in Hopfield-type networks, i.e. the storage capacity in terms of numbers of stored attractors per available synapse increases with pruning (Sompolinsky, 1986; Derrida et al., 1987). Another line of work has characterized the statistics of synaptic connectivity in networks that optimize the robustness of fixed-point attractor states or sequences (Kohler and Widmaier, 1991; Brunel et al., 2004; Brunel, 2016; Zhang et al., 2019). In networks of excitatory and inhibitory neurons increasing the robustness of attractors led to networks with synaptic connectivity distributions that were sparse (i.e. many absent connections) but strong (i.e. the connections that remained were larger) (Kohler and Widmaier, 1991; Brunel, 2016; Zhang et al., 2019). It was also shown that the measured distribution of synaptic strengths in cortex was consistent with the predictions of the theory (Brunel, 2016). Thus, this work suggested that robust attractor dynamics lead to sparsely connected networks with fewer but stronger connections. Our work further extends these results by showing that pruning of weak connections in networks with sequential Hopfield connectivity leads to large attractor basins for strong memories, while having minimal effects or eliminating memories that are weakly encoded.

Although we have reasonable network models for the computations that underlie cognition and learning (Sussillo, 2014), like the models we have explored here, less is known about the specific neural systems in which these computations are implemented. For example, OCD is thought to reflect disordered computation in cortical-basal ganglia-thalamocortical circuits (Milad and Rauch, 2012; Ahmari and Dougherty, 2015; Sha et al., 2020). Whether OCD symptomatology reflects disordered computation in only one component of this circuitry, that is then propagated through the rest of the circuitry, or deficits in multiple nodes of the circuit, is unclear. Neurophysiology experiments in monkeys carrying out reinforcement learning tasks have consistently shown that task variables related to affective learning have similar representations across nodes of CBTC circuits (Tang et al., 2022; Tang et al., 2024). Information about task variables appears to propagate throughout the circuit in 10s of milliseconds, consistent with known synaptic delays.

We have motivated our hypothesis from the perspective of strong memories that are embedded in networks before pruning, that subsequently come to dominate the attractor landscape after pruning. During adolescence, when weak synapses are pruned and stronger synapses are further strengthened (Chung et al., 2017; Li et al., 2017), emotionally intense memories come to dominate the attractor landscape, and less intense memories are pruned away. These dynamics can lead to treatment resistance, or the presence of unwanted, intrusive thoughts that are difficult to suppress. This can account for disorders including PTSD that follows childhood abuse and neglect, the effects of rumination in depression, early exposure to drugs of abuse in addiction (Anthony and Petronis, 1995; Chambers et al., 2003), and some forms of OCD in which the compulsions or rituals are linked to the obsessions or intrusive thoughts in a straightforward way, like handwashing compulsions with contamination obsessions. This hypothesis can also link early life stress or adverse childhood events to the increase of the prevalence of psychiatric disorders as the early life stress can embed strong memories (McLaughlin et al., 2019; Aloi et al., 2024) that become dominant through synaptic pruning.

It is not as immediate to account for OCD in which the compulsions do not have a reasonable link to the obsession. These cases may have some overlap with delusional ideation(O'Dwyer and Marks, 2000). It has been suggested that chaotic activity in dopamine neurons can lead to irrational associations (Kapur et al., 2005). In effect, if the neuromodulator component of three-factor learning is dissociated from coherent events which normally give rise to intense emotions, bizarre associations could be formed and embedded in neural systems. Our hypothesis makes the strong claim that persistent ritualized behavior, or intrusive thoughts and memories that are difficult to suppress, are driven by strong attractor dynamics. And pruning and myelination leads to networks that can be dominated by fewer but stronger dynamical attractors. The mechanism that gives rise to the strong embedded memories may in some cases be determined by coherent life events and in others by stochastic but strong activity in neuromodulator systems that embeds attractors that are only weakly associated with coherent life events, but nevertheless lead to embedding strong patterns of activity in synaptic weights. Additional work will be necessary to better understand these mechanisms.

Ketamine and psilocybin, which are novel approved or under consideration treatments for depression and other psychiatric disorders, have been shown to increase spine density and therefore excitatory connections, in cortex (Savalia et al., 2021; Shao et al., 2021). Electroconvulsive therapy (ECT) may also increase spine density, because ECT induces seizures which could drive spine formation through activity dependent mechanisms. These treatments may alleviate clinical symptoms by increasing the capacity for plasticity and therefore opening a window for other treatments like cognitive behavioral therapy, to work. However, it is also possible that increasing spine density may lead to clinical improvements by decreasing the strength of dynamical attractors. Adding random spines to our pruned networks makes the sequential dynamics less robust (data not shown). As we have shown in the present paper, and in a previous study (Averbeck, 2022), pruning increases the basin of attraction of dynamical attractors. While this can lead to improvements in cognition when the attractors represent beneficial patterns of activity, it can lead to disorders when the attractors represent negative emotional states.

## Figures and Tables

**Table 1.** Parameter values used in spiking network simulations.

Parameter	Value
$J^{S}$	6.3
$\tau_{m}^{E}$	0.010 s
$\tau_{S}^{E}$	0.020 s
$V_{\mathrm{threshold}}$	20 mv
$V_{\mathrm{reset}}$, $V_{\mathrm{rest}}$	0 mv
$V_{\mathrm{reversal}}^{E}$	−10 mv
${\tau^{I}}_{m}$	.002 s
${\tau^{I}}_{s}$	.005 s
$\sigma^{E}$	5.0
$\sigma^{I}$	5.0
$p^{EE}$	0.08
$p^{EI}$	0.04
$p^{IE}$	0.04
$p^{EEx}$	0.04
$x_{f}$, $x_{g}$	1.5
$q_{f}$	0.8
$q_{g}$	0.932
